# Seasonal flooding regime and ecological traits influence genetic structure of two small rodents

**DOI:** 10.1002/ece3.1336

**Published:** 2014-11-30

**Authors:** Rita Gomes Rocha, Eduardo Ferreira, Carlos Fonseca, Juliana Justino, Yuri Luiz Reis Leite, Leonora Pires Costa

**Affiliations:** 1 CESAM and Departamento de Biologia, Universidade de AveiroCampus de Santiago, Aveiro, 3810-193, Portugal; 2 Laboratório de Mastozoologia e Biogeografia, Departamento de Ciências Biológicas, Centro de Ciências Humanas e Naturais, Universidade Federal do Espírito Santo AvGoiabeiras, 29075-910, Vitória, ES, Brazil; 3 Núcleo de Genética Aplicada à Conservação da Biodiversidade, Departamento de Ciências Biológicas, Universidade Federal do Espírito SantoAv. Marechal Campos 1468, Maruípe, Vitória, 29043-900, Espírito Santo, Brazil

**Keywords:** Amazonia-Cerrado ecotone, Araguaia river, Cricetidae, mitochondrial DNA, Rodentia

## Abstract

Although codistributed species are affected by the same abiotic factors, such as rivers and seasonal flooding regimes, ecological traits, such as locomotion habits and habitat preferences, may also influence differences in levels of genetic diversity and differentiation. We examined population genetic structure and diversity of *Hylaeamys megacephalus* and *Oecomys* aff*. roberti*, two cricetid rodent species from the mid-Araguaia River in central Brazil, using mitochondrial DNA sequence data. Specifically, we aim to test whether the Araguaia River acts as a barrier to the gene flow of these two species and to assess how ecological traits, such as locomotion habits and habitat preferences, may influence differences in levels of genetic diversity and differentiation. As both species occur in flooded forests, neither showed genetic differences related to river banks. *Oecomys* aff*. roberti* showed stronger population structure that appears to be associated with isolation by distance. This arboreal species maintained stable populations in the Araguaia River, while the terrestrial *H. megacephalus* was more affected by seasonal floods, resulting in a genetic signature of population expansion. Our initial predictions were largely supported by our results given that locomotion habits and habitat preferences of each species appears to have played a role on the genetic structure of these two sympatric rodent species.

## Introduction

Genetic differentiation is the result of the interaction of both historical and ongoing evolutionary processes (e.g., Moritz et al. 2000; Patton et al. 2000). Landscape features such as rivers and mountains have been considered important factors in shaping the genetic structure of Neotropical species (Antonelli et al. 2010; Leite and Rogers 2013). However, the ability to disperse across these landscape features and to recover from bottlenecks is also related with species ecology and life history (Matocq et al. 2000). Indeed, dispersal tendencies, habitat preferences, life span, and mating system and success determine population sizes and gene flow rates among populations, which in turn will determine levels of genetic diversity and differentiation (Allendorf and Luikart 2007).

The Araguaia River in central Brazil is one of the main drainage basins of the Cerrado biome and lies in the transition to Amazonia (Latrubesse et al. 2009). This river represents a relatively stable geographical barrier due to its anabranching pattern, consisting of multiple channels separated by vegetated semipermanent alluvial islands, with low sinuosities (Latrubesse 2008), which are expected to limit gene flow between population of forest dwellers from opposite river banks (e.g., Aleixo 2004; Bates et al. 2004; Antonelli et al. 2010; Faria et al. 2013; Leite and Rogers 2013). River geomorphology has been argued as a possible cause of differences in the genetic differentiation of populations by river bank, and anabranching rivers like the Araguaia are probably stronger barriers than meandering rivers, such as the Juruá (e.g., Moritz et al. 2000; Patton et al. 2000; and Antonelli et al. 2010). Indeed, previous studies have shown that the Araguaia is an important barrier to sister species of the climbing rats of the genus *Rhipidomys* (Bonvicino et al. 2008; Rocha et al. 2011), but it is not known to what extent this finding is consistent across other cricetid rodents with different ecological traits.

Here, we examine two species, *Hylaeamys megacephalus* and *Oecomys* aff*. roberti*, that were the two most abundant cricetid rodents captured in the mid-Araguaia River basin (Rocha et al. 2011). *Hylaeamys megacephalus* is a terrestrial and generalist species with high habitat tolerance (Emmons and Feer 1997; Percequillo et al. 2008), occurring in both flooded and unflooded forests of the Araguaia River (Rocha et al. 2011). *Oecomys* aff*. roberti* (treated as *Oecomys* sp. in Rocha et al. 2011) is so far known only from the Araguaia River, where it presents arboreal habits and prefers flooded forests (Rocha et al. 2011; Ramos Pereira et al. 2013), like its closely related congener *O. roberti* in the Juruá River (Patton et al. 2000).

We used two mitochondrial molecular markers (cytochrome B and D-loop) to compare population genetic structure and diversity of these two cricetid rodent species from the mid-Araguaia River in central Brazil. Specifically, we aim to test whether the Araguaia River acts as a barrier to the gene flow of these two species and to assess how ecological traits, such as locomotion habits and habitat preferences, may influence differences in levels of genetic diversity and differentiation. These two similar-sized species co-occur along the Amazonia-Cerrado ecotone, but they show distinct ecological traits, leading to different predictions regarding levels of genetic diversity and differentiation (Table 1). As both species occur in flooded forests, we hypothesize that both will exhibit low differentiation across river banks, showing haplotype sharing between river banks and islands. However, *H. megacephalus* will likely present lower overall genetic differentiation than *O.* aff*. roberti*, as the former is a more generalist species in terms of habitat. We also hypothesize that the ground-dwelling species, *H. megacephalus*, is more prone to reveal population size fluctuations following seasonal flooding regime, while the arboreal species, *O.* aff*. roberti*, is more likely to maintain stable populations in the Araguaia River throughout the year (Table 1).

**Table 1:** Summary of ecological traits and genetic predictions for *Hylaeamys megacephalus* and *Oecomys* aff*. roberti* in mid-Araguaia River, central Brazil

Species	Ecological traits	Genetic predictions
*H. megacephalus*	Terrestrial, more prone to local extinction due to seasonal flooding regime	Unimodal mismatch distribution; evidence of population expansion in neutrality tests; proportionally less haplotypes in islands and shared between river banks and islands due to higher rate of extinction
	Habitat generalist	Weaker genetic structure (lower *F*_ST_) and no evidence of isolation by distance
	Occurs in both seasonally flooded and unflooded forests	Low differentiation across river banks, shared haplotypes between river banks and islands
*O*. aff. *roberti*	Arboreal, less prone to local extinctions due to seasonal flooding regime	Multiple peaks in mismatch distribution; no evidence of population expansion in neutrality tests; proportionally more haplotypes present in islands and shared between river banks and islands due to lower rate of extinction
	Habitat specialist	Stronger genetic structure (higher *F*_ST_) and isolation by distance
	Prefers seasonally flooded forests	Low differentiation across river banks, shared haplotypes between river banks and islands

## Methods

### Study area and sample collecting

**Figure 1:**
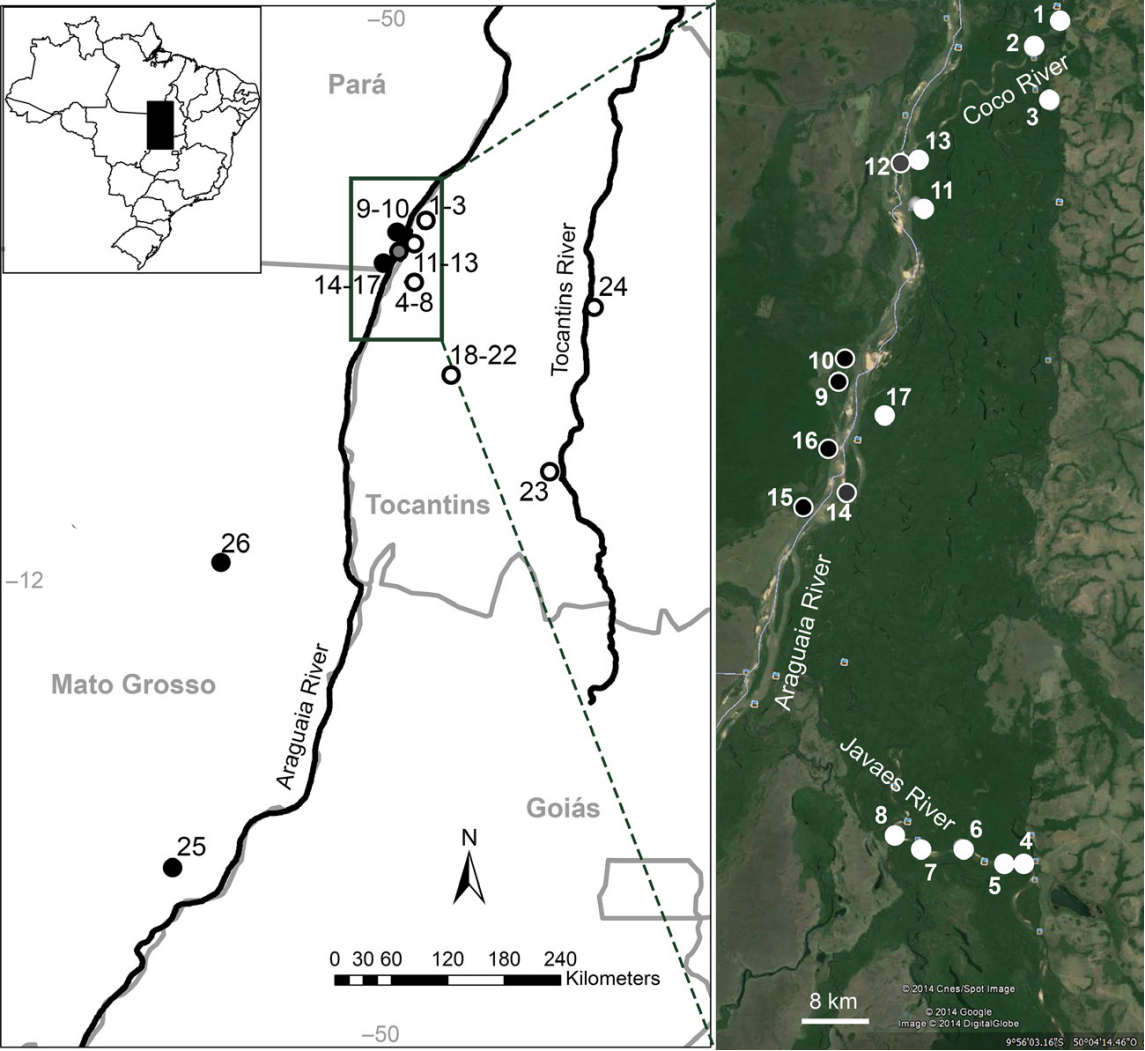
Study area in the mid-Araguaia River basin. Location of study area in Brazil (upper left corner); Map including all the sampling localities (middle) and detail of sampling points in the Araguaia River and its tributaries (right, source: Google Earth). Sampling points in white corresponds to the eastern river bank, in black to the western river bank, and in gray to the islands of the Araguaia River.

Specimens of *Hylaeamys megacephalus* and *Oecomys* aff*. roberti* were collected from 26 sampling points in the mid-Araguaia River and its surroundings (Fig. 1, see gazetteer in Supplementary material for locality names and geographical localization). The Araguaia River is an anabranching river consisting of multiple channels separated by vegetated semipermanent alluvial islands excised from preexisting floodplain or formed by within-channel or deltaic accretion (Narson and Knighton 1996*;* Latrubesse 2008). The landscape is characterized by a mosaic of seasonally flooded and unflooded gallery forests located along the margins of the streams and channels. In well-drained areas, vegetation physiognomies more typical of the Cerrado occur (Oliveira-Filho and Ratter 2002). The climate is tropical, with a rainy season lasting from October to April and a dry season from May to September (INMET 2011).

Sampling was carried out between June 2007 and November 2008, using a standardized trapping protocol to sample small nonvolant mammals in upland and floodplain gallery forests. Live traps (Sherman and Tomahawk) and pitfalls (30 L and 60 L) were set in each sampling point. Three sampling periods averaging seven nights (between five and nine nights) were performed in each sampling point (see Rocha et al. 2011 for detailed information on habitat, sampling, and specimens). Tissue samples have been deposited at Universidade Federal do Espírito Santo (UFES), Vitória, Brazil.

### DNA extraction and amplification

DNA was extracted from liver and ear tissue samples preserved in ethanol using the salt-extraction method (Bruford et al. 1992). An 801-bp fragment of the cyt B was amplified by polymerase chain reaction (PCR) using the primers MVZ05 and MVZ16 (Smith and Patton 1993) for both species. 860-bp fragments of D-loop were amplified by PCR using the primers L15774M and H651B (Fumagalli et al. 1997) for *H. megacephalus*. 496-bp fragments of D-loop were amplified by PCR using the primers L0 and E3 (Douzery and Randi 1997; Huchon et al. 1999) for *O.* aff*. roberti*. Amplifications were performed using the following PCR profiles: (1) cyt B: initial denaturation at 94°C for 5 min, followed by 39 cycles with denaturation at 94°C for 30 s, annealing at 48°C for 45 s, polymerization at 72°C for 45 s, and a final extension at 72°C for 5 min; (2) D-loop: initial denaturation at 94°C for 2 min, followed by 35 cycles with denaturation at 94°C for 30 s, annealing at 54°C (*H. megacephalus*) or 45°C (*O.* aff*. roberti*) for 90 s, polymerization at 68°C for 1 min, and a final extension at 72°C for 10 min. Mitochondrial fragments were purified using ExoSap-IT® (USB Corporation, Cleveland Ohio, US) and sequenced using an automatic sequencer ABI 3130-XL (Perkin Elmer, Applied Biosystems, Foster City, California), with the above-listed primers.

Sequence alignment was performed using CLUSTALW algorithm implemented in MEGA version 5 (Tamura et al. 2011), with posterior manual edition. All sequences generated in this study have been deposited in GenBank (Table 2).

**Table 2:** List of the cytochrome B and D-loop haplotypes (h) of *Hylaeamys megacephalus* and *Oecomys* aff*. roberti*, with frequencies (*F*), sampling localities, and GenBank accession numbers. Sampling locality numbers correspond to those mapped in Figure 1. See Gazetteer in Supplementary material for locality names and geographical localization

	cyt B				D-loop			
		
Taxon	h	*F*	Locality	GenBank accession	h	*F*	Locality	GenBank accession
*H. megacephalus*	HHc1	12	4, 13, 15, 16	KP027738	HHd1	9	4, 13, 15, 16	KP027770
	HHc2	1	8	KP027739	HHd2	1	8	KP027771
	HHc3	1	1	KP027740	HHd3	1	1	KP027772
	HHc4	3	5, 10, 14	KP027741	HHd4	3	5, 10, 14	KP027773
	HHc5	6	5, 6, 16, 26	KP027742	HHd5	3	5, 6	KP027774
	HHc6	1	5	KP027743	HHd6	1	5	KP027775
	HHc7	1	5	KP027744	–			
	HHc8	3	6, 8, 22	KP027745	HHd7	1	6	KP027776
	HHc9	2	5, 17	KP027746	–			
	HHc10	1	2	KP027747	HHd8	1	2	KP027777
	–				HHd9	1	13	KP027778
	–				HHd10	1	16	KP027779
	HHc11	1	16	KP027748	HHd12	1	16	KP027781
	HHc12	1	21	KP027749	HHd11	1	21	KP027780
	HHc13	4	21, 23	KP027750	HHd13	2	21	KP027782
	HHc14	1	21	KP027751	HHd14	1	21	KP027783
	–				HHd15	1	22	KP027784
	HHc15	1	19	KP027752	HHd16	1	19	KP027785
	HHc16	1	16	KP027753	HHd17	2	15, 16	KP027786
	–				HHd18	1	17	KP027787
	HHc17	4	15, 25, 26	KP027754	HHd19	1	25	KP027788
	HHc18	2	23	KP027755	–			
	HHc19	1	23	KP027756	–			
	HHc20	1	23	KP027757	HHd20	1	23	KP027789
	–				HHd21	1	25	KP027790
	–				HHd22	1	26	KP027791
	HHc21	1	26	KP027758	HHd23	2	25, 26	KP027792
*O.* aff*. roberti*	HOc1	14	1, 2, 3, 7, 8, 10, 12, 13, 14	HM594599	HOd1	3	1, 6, 14	KP027759
	HOc2	5	1, 2, 12, 13	HM594598	HOd2	6	1, 2, 12, 13, 22	KP027760
	–				HOd3	12	2, 3, 8, 9, 10, 13	KP027761
	–				HOd4	1	3	KP027762
	HOc3	5	1, 11, 13	HM594602	HOd5	6	1, 11, 13	KP027763
	–				HOd6	9	11, 12, 13	KP027764
	HOc4	1	6	KP027736	–			
	HOc5	1	2	HM594601	HOd7	1	2	KP027765
	–				HOd8	1	17	KP027766
	HOc6	1	22	KP027737				
	HOc7	6	18, 19, 20, 22	HM594600	HOd9	1	19	KP027767
	–				HOd10	4	18, 20, 22	KP027768
	HOc8	1	23	HM594596	–			
	HOc9	1	23	HM594595	HOd11	1	23	KP027769

### Genetic diversity indices and population genetic structure

Number of haplotypes and polymorphic sites, haplotype and nucleotide diversity values were estimated with DnaSP v.5 (Librado and Rozas 2009) for both molecular markers from each species. Phylogeographic relationships among haplotypes (concatenated cyt B and D-loop *loci*) from both species were estimated through Bayesian inference (BI) in MrBayes v.3.1.2 (Ronquist and Huelsenbeck 2003). The model of nucleotide substitution was selected using MrModeltest (Nylander 2004). Haplotype trees were sampled every 100 of 10^7^ generations until Markov chain became stationary, that is, when standard deviation of split frequencies was below 0.01 (Ronquist et al. 2009). A 50% majority rule consensus tree was obtained after “burn-in” of 25% of the sample points to generate Bayesian posterior probabilities (BPP). Trees were then edited in FigTree v1.4 (available at http://tree.bio.ed.ac.uk/software/figtree/).

Median-joining (MJ) networks were produced for both markers separately using NETWORK (Bandelt et al. 1999) and using only variable nucleotide sites. We divided the samples into three subpopulations based on geography (one from each river bank and another from the islands) to test the role of the river in population structuring.

Spatial analyses of molecular variance were performed for both markers separately using SAMOVA 1.0 (Dupanloup et al. 2002) to identify groups of populations, which accounted for genetic differentiation and geographic distances. Analyses were performed for *K* = 2 to 10 to select the optimal number of groups (*K*), with the 26 sampling points for each species. *K* was selected based on the maximum (or plateau) value of fixation index *F*_CT_ (variance among groups relative to the total variance) and on the minimum number of groups with single populations.

The genetic differentiation between sampling points was assessed with pairwise *F*_ST_ estimates, using ARLEQUIN 3.5.1.3 (Excoffier and Lischer 2010). The *P*-value was estimated using 10,000 permutations. Isolation by distance was assessed using a simple Mantel test. The correlation between matrices of pairwise estimates of genetic differentiation (*F*_ST_) and matrix of geographic distances was estimated with the Mantel test, using Isolation by Distance web service v.3.23 (Jensen et al. 2005).

### Demographic history

Neutrality and demographic history were evaluated for both markers separately from the entire sample distribution of each species using DnaSP v.5 (Librado and Rozas 2009). Populations under expansion are expected to exhibit smooth and unimodal mismatch distributions, while populations at demographic equilibrium are characterized by ragged and erratic mismatch distributions (Harpending 1994). Deviations from the sudden population expansion model were further tested using the Harpending's raggedness statistics, which quantify the smoothness of the observed distribution (Harpending 1994). We also used statistics to detect demographic events based on frequency spectrum of mutations, Tajima's (1989) *D* and Ramos-Onsins and Rozas's (2002) *R*_2_, and based on haplotype frequencies, Fu's (1997) *Fs*. Coalescence simulations with 1000 replicates were applied to determine the *P*-value of each statistics.

## Results

### Genetic diversity indices

Partial sequences of cyt B and D-loop were obtained from 49 and 38 individuals of *H. megacephalus*, respectively, resulting in 21 haplotypes and 36 polymorphic sites for cyt B, and 23 haplotypes and 79 polymorphic sites for D-loop (Table 3). Partial sequences of cyt B and D-loop were obtained from 35 and 45 individuals of *O*. aff. *roberti*, respectively, resulting in nine haplotypes and 16 polymorphic sites for cyt B, and 11 haplotypes and 18 polymorphic sites for D-loop (Table 3). Haplotype and nucleotide diversities were relatively higher for D-loop than cyt B for both species (Table 3).

**Table 3:** Number of individual sequences (*n*), base pairs (bp), haplotypes (h), polymorphic sites (S), haplotype diversity (Hd), nucleotide diversity (*π*), raggedness statistics (*r*), and deviation from neutrality tests (*D*, *Fs*, *R*_2_)

Species	Marker	*n*	Bp	h	S	Hd	*π*	*r*	D	*F*s	*R*_2_
*H. megacephalus*	cyt B	49	801	21	36	0.914	0.006	0.035	−1.269	−6.042*	0.063
	D-loop	38	860	23	79	0.936	0.016	0.015	−1.252	−2.483	0.082
*O.* aff. *roberti*	cyt B	35	801	9	16	0.788	0.005	0.153	−0.035	0.785	0.122
	D-loop	45	460	11	18	0.858	0.007	0.101	0.172	0.280	0.115

* Significant values (*P* < 0.05).

### Population genetic structure

**Figure 2:**
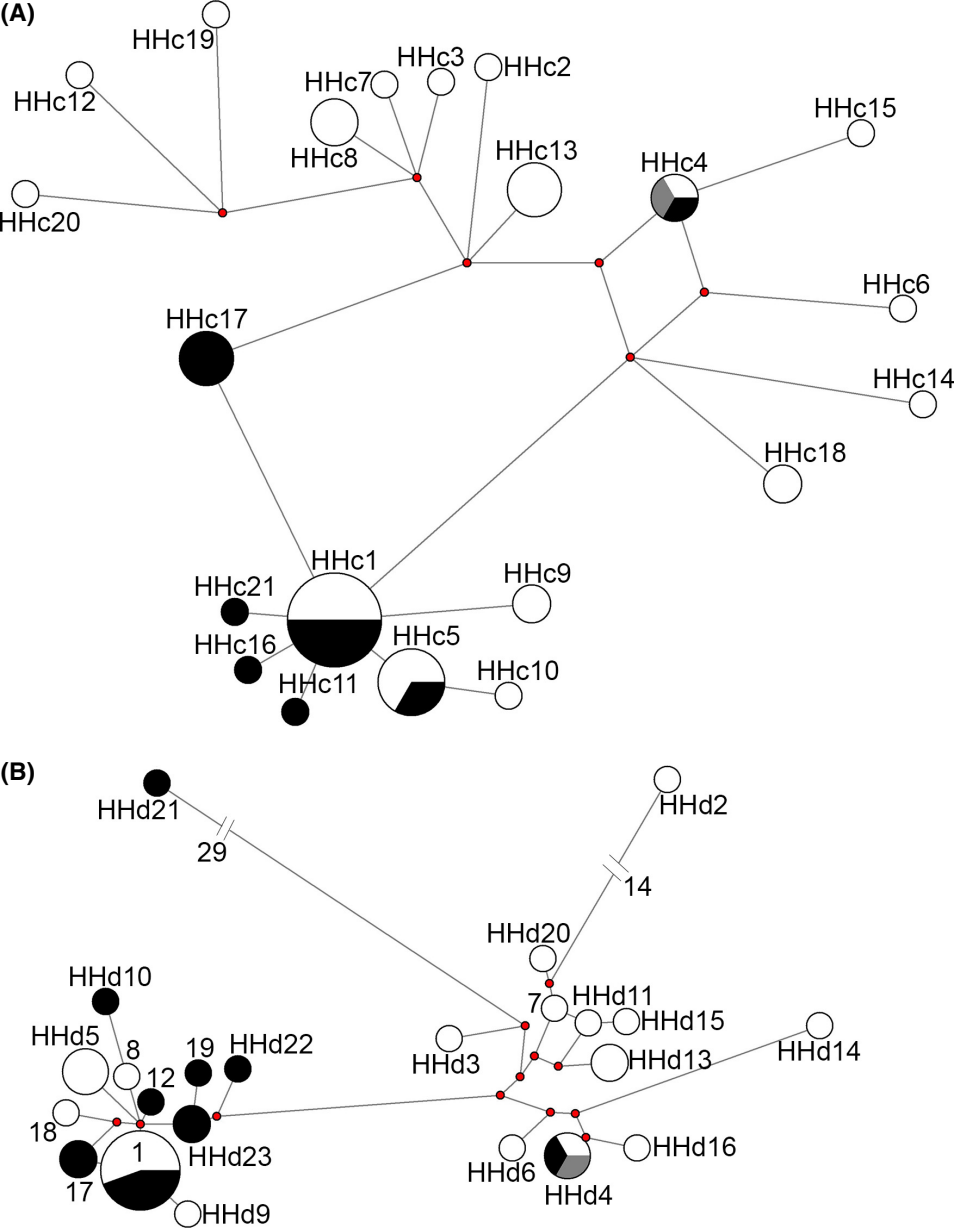
Median-joining networks of cyt B (A) and D-loop (B) sequences of *Hylaeamys megacephalus*. Length of connection branches corresponds to the nucleotide substitutions; size of the circles is proportional to the number of individuals sharing each haplotype (for details see Table 1); white corresponds to the eastern river bank, black to the western river bank, and gray to the islands of the Araguaia River.

**Figure 3:**
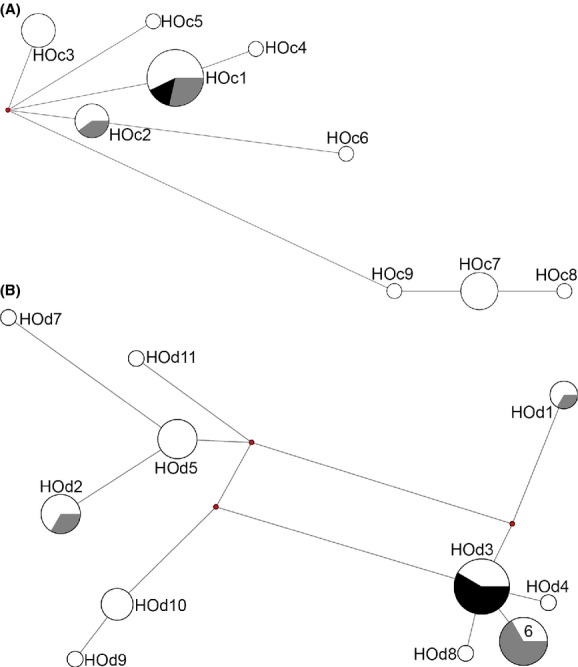
Median-joining networks of cyt B (A) and D-loop (B) sequences of *Oecomys* aff*. roberti*. Length of connection branches corresponds to the nucleotide substitutions; size of the circles is proportional to the number of individuals sharing each haplotype (for details see Table 1); white corresponds to the eastern river bank, black to the western river bank, and gray to the islands of the Araguaia River.

Median-joining networks from both markers revealed intraspecific haplotype sharing across the mid-Araguaia River and also on islands (Figs 2 and 3). Two *H. megacephalus* cyt B haplotypes (HHc1 and HHc5) and one D-loop haplotype (HHd1) were shared by specimens from both river banks; and one haplotype of each marker (HHc4 and HHd4) was also shared with specimens from the islands (Fig. 2). One *O.* aff. *roberti* cyt B haplotype (HOc1) was shared by specimens from both river banks, along with specimens from the islands; one cyt B haplotype (HOc2) and three D-loop haplotypes (HOd1, HOd2, and HOd6) were shared by specimens from the eastern river bank and the islands; and one D-loop haplotype (HOd3) was shared by specimens from both river banks (Fig. 3). Bayesian inference (BI) trees obtained for concatenated haplotypes resulted in a basal polytomy (Fig. S1). The BI tree for *H. megacephalus* lacked a clear geographic structure and no haplogroups could be identified (Fig. S1A). In *O.* aff. *roberti*, on the other hand, two haplogroups were identified, but without clear geographic structure (Fig. S1B).

Using SAMOVA, we found three (*K* = 3) and four (*K* = 4) groups of populations in both species based on cyt B and D-loop, respectively (Table 4). These groups were not geographically consistent, but two main patterns were observed for both species. Sampling points from the mid-Araguaia River are consistently grouped (*H. megacephalus*: SHc3 and SHd4; *O*. aff. *roberti*: SOc2, SOd1, and SOd4), and sampling points from Uburu and Tocantins were grouped separately (*H. megacephalus*: SHd3; *O*. aff. *roberti*: SOc1 and SOd2), with the exception of groups SHc1, SHc2, and SHd1 of *H. megacephalus* and groups SOc2 and SOd3 of *O*. aff. *roberti*, which cluster sampling points from both regions (Table 4).

**Table 4:** Spatial analyses of molecular variance (SAMOVA) results for both species based on cytochrome B and D-loop markers

Species	Marker	*K*	*F*_CT_	Group	Sampling points
*H. megacephalus*	cyt B	3	0.50**	SHc1	1, 8, 10, 21, 22, 23
				SHc2	14, 19
				SHc1	2, 4, 5, 6, 13, 15, 16, 17
	D-loop	4	0.51**	SHd1	1, 10, 14, 19
				SHd2	8
				SHd3	21, 22, 23
				SHd4	2, 4, 5, 6, 13, 15, 16, 17, 25, 26
*O.* aff. *roberti*	cyt B	3	0.75**	SOc1	18, 19, 20, 22, 23
				SOc2	1, 2, 3, 6, 7, 8, 10, 12, 13, 14
				SOc3	11, 24
	D-loop	4	0.48**	SOd1	6, 14
				SOd2	19, 20, 22, 18
				SOd3	1, 2, 11, 13, 23
				SOd4	3, 7, 8, 9, 10, 12, 17

** Significant values (*P* < 0.001).

Most *F*_ST_ values were not significant in either species (Table S1 and S2). Only sampling points 21 and 23 revealed moderate-to-high levels of differentiation in *H. megacephalus*, and sampling points 12 and 20 revealed high levels of differentiation in *O*. aff. *roberti* (Table S1 and S2).

We found no correlation between genetic and geographic distances in *H. megacephalus* (cyt B: *r* = 0.001, *P* = 0.545; D-loop: *r* = −0.19, *P* = 0.828), refuting a scenario of isolation by distance; but a positive correlation was found in *O*. aff. *roberti* (cyt B: *R* = 0.364, *P* = 0.001; D-loop: *R* = 0.318, *P* = 0.002), indicating isolation by distance.

### Demographic history

**Figure 4:**
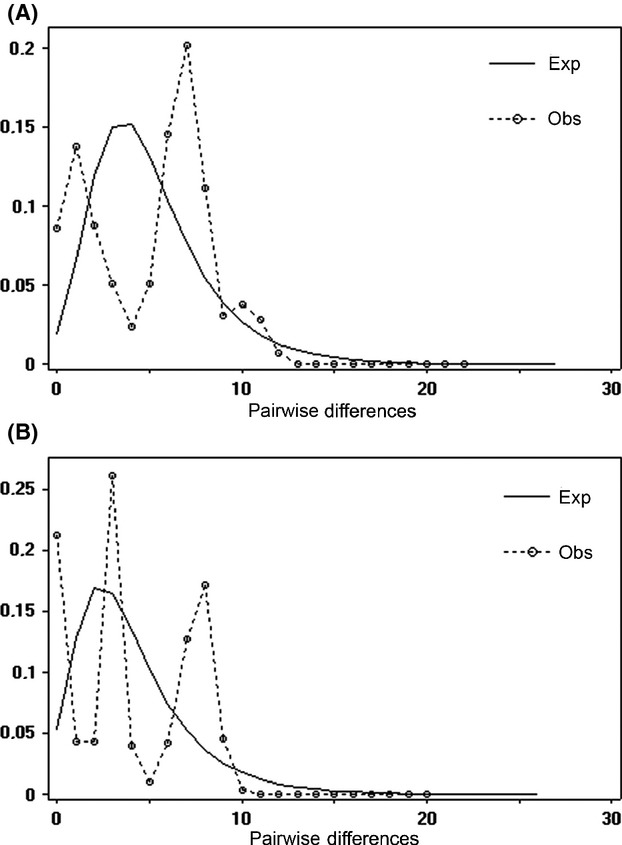
Mismatch distribution graphs of *Hylaeamys megacephalus* (A) and *Oecomys* aff*. roberti* (B). The dashed curves indicate the observed frequency distribution of pairwise differences; and solid curves indicate the distribution that would be expected under a population growth–decline model. ⊖ and *τ* were estimated using DnaSP software. *Hylaeamys megacephalus*: ⊖ = 2.261 and *τ* = 2.789 and *Oecomys* aff*. roberti*: ⊖ = 2.338 and *τ* = 1.746.

We found no evidence of population expansion in the mismatch distributions of either species (Fig. 4). Consistently, the observed raggedness values were not significantly lower than the expected in an expansion model for both markers (Table 3). The only significant negative value was Fu's *Fs* for the cyt B dataset of *H. megacephalus* (Table 3). All other neutrality tests showed nonsignificant values (Table 3), therefore rejecting the population expansion model.

## Discussion

Although previous studies have shown that the Araguaia River is an important barrier to some small mammals (Rocha 2011; Rocha et al. 2011; Faria et al. 2013), we found no evidence of this riverine barrier on the two rodent species analyzed. Molecular phylogenies showed no geographic structure, and there are shared haplotypes across river banks and throughout the islands of the Araguaia River. This pattern of haplotype sharing indicates that these islands are important stepping stones facilitating gene flow between river banks.

Because the Araguaia River has not been acting as an effective abiotic barrier to gene flow among populations of *O.* aff. *roberti* and *H. megacephalus*, differences in genetic structure and diversity between these two species may be related to biotic factors, like their ecological traits. Differences in overall patterns of genetic diversity at a local scale can be explained by the combination of historical and ongoing processes of differentiation, but also by the ecological traits of each species (Patton et al. 1996). For example, Matocq et al. (2000) compared the genetic structure of two sympatric and ecologically distinct echimyid rodents, *Proechimys steerei* and *P. simonsi,* and found that the former, which inhabits the seasonally flooded forest, exhibits high gene flow, while the latter, which occupies upland, unflooded habitats, shows little or no evidence of ongoing gene flow among populations.

*Hylaeamys megacephalus* is a generalist species with high habitat tolerance (Percequillo et al. 2008), thus allowing higher gene flow among populations, even across long distances. The lack of correlation between genetic and geographic distances, even for samples that are very distant (e.g., sampling points 25 and 26), corroborates a scenario of gene flow instead of isolation by distance for this species. In addition, *H. megacephalus* is terrestrial, and we therefore expect population declines during the flooding season, followed by expansions in the dry season when more ground habitat is available. Although an erratic mismatch distribution may indicate a population in equilibrium, significant negative values in neutrality tests, such as shown by the cyt B data from *H. megacephalus*, as well as star-like genealogies showing very short and very long branch lengths, suggest population expansion (Ray et al. 2003). Maruyama and Fuerst (1985) also argue that population bottlenecks followed by rapid expansion increase the number of rare alleles in natural populations. Indeed, a higher number of rare haplotypes were recorded for *H. megacephalus* in the Araguaia River. Additionally, the maintenance of high haplotype diversity of populations that experience demographic declines during the seasonal floods is possible due to high reproductive potential of the species, allowing rapid expansion after the flooding, and to the intermixing of the surviving populations (Matocq et al. 2000).

In contrast, *O.* aff. *roberti* is more specialized, showing arboreal habits, and preference for flooded forests, thus influencing gene flow among distant populations and resulting in higher genetic differentiation. Although most *F*_ST_ values were not significant, this pattern is corroborated by a significant positive correlation between genetic and geographic distances, suggesting isolation by distance. Additionally, *O*. aff. *roberti* is less prone to be affected by the flooding regime in the Araguaia River because of its arboreal habits. Indeed, *O.* aff. *roberti* showed nonsignificant values for the neutrality tests and also an erratic mismatch distribution, which indicate stable rather than expanding populations.

When comparing several small mammal species within Araguaia River basin, we corroborate previous hypothesis by Moritz et al. (2000), which pointed out that upland forest specialists are more prone to exhibit riverine diversification than specialists of floodplain forests. Indeed, both *H. megacephalus* and *O.* aff. *roberti* are well distributed across flooded forests, while other sympatric small mammals, such as the mouse opossum *Marmosa murina* and the climbing rats *Rhipidomys* spp., are upland specialists (Patton et al. 2000; Rocha et al. 2011) genetically differentiated by the Araguaia River banks (see Rocha 2011; Rocha et al. 2011 and Faria et al. 2013).

Our initial genetic predictions based on ecological traits were largely supported by the results of both *H. megacephalus* and *O.* aff. *roberti* from the mid-Araguaia River. We showed that species with different ecological adaptations have different responses to physical and temporal events, and such events can leave fingerprints on the genetic diversity and differentiation patterns. Although the two rodent species co-occurring in flooded forests are not genetically structured by river bank, the fact that *H. megacephalus* is terrestrial and *O*. aff. *roberti* is arboreal made them more or less prone to local extinction due to the seasonal flooding regime of the Araguaia River. Additionally, differences in habitat tolerance are also important in differentiating populations from distant locations. Further comparative studies will help elucidate in which ways differences in ecological traits may influence the geographic distribution of genetic diversity. The combination of molecular and ecological studies is a powerful tool in understanding evolutionary processes that determine species diversification in the Neotropics.

## Supporting Information

**Figure S1.** Bayesian inference tree of concatenated haplotypes of *Hylaeamys megacephalus* (A) and *Oecomys* aff. *roberti* (B). **Table S1.** Pairwise *F*_ST_ values based on cyt B sequences (below diagonal) and d-loop sequences (above diagonal) of *H. megacephalus*. *F*_ST_ values in bold were significantly different from zero. **Table S2.** Pairwise *F*_ST_ values based on cyt B sequences (below diagonal) and d-loop sequences (above diagonal) of *O.* aff. *roberti*. *F*_ST_ values in bold were significantly different from zero. **Data S1.** Gazetteer List 26 collecting localities of *Oecomys* aff. *roberti* and *Hylaeamys megacephalus*, sorted by Brazilian states.
