# Supplementary material for: Seasonal flooding regime and ecological traits influence genetic structure of two small rodents
Source: Ecol Evol. 2014 Nov 30;4(24):4598–608. doi: 10.1002/ece3.1336 (PMC4278813; doi:10.1002/ece3.1336)
Supplement: Supplementary file 1 [file ece30004-4598-sd1.docx]

**Supplementary Material**

**
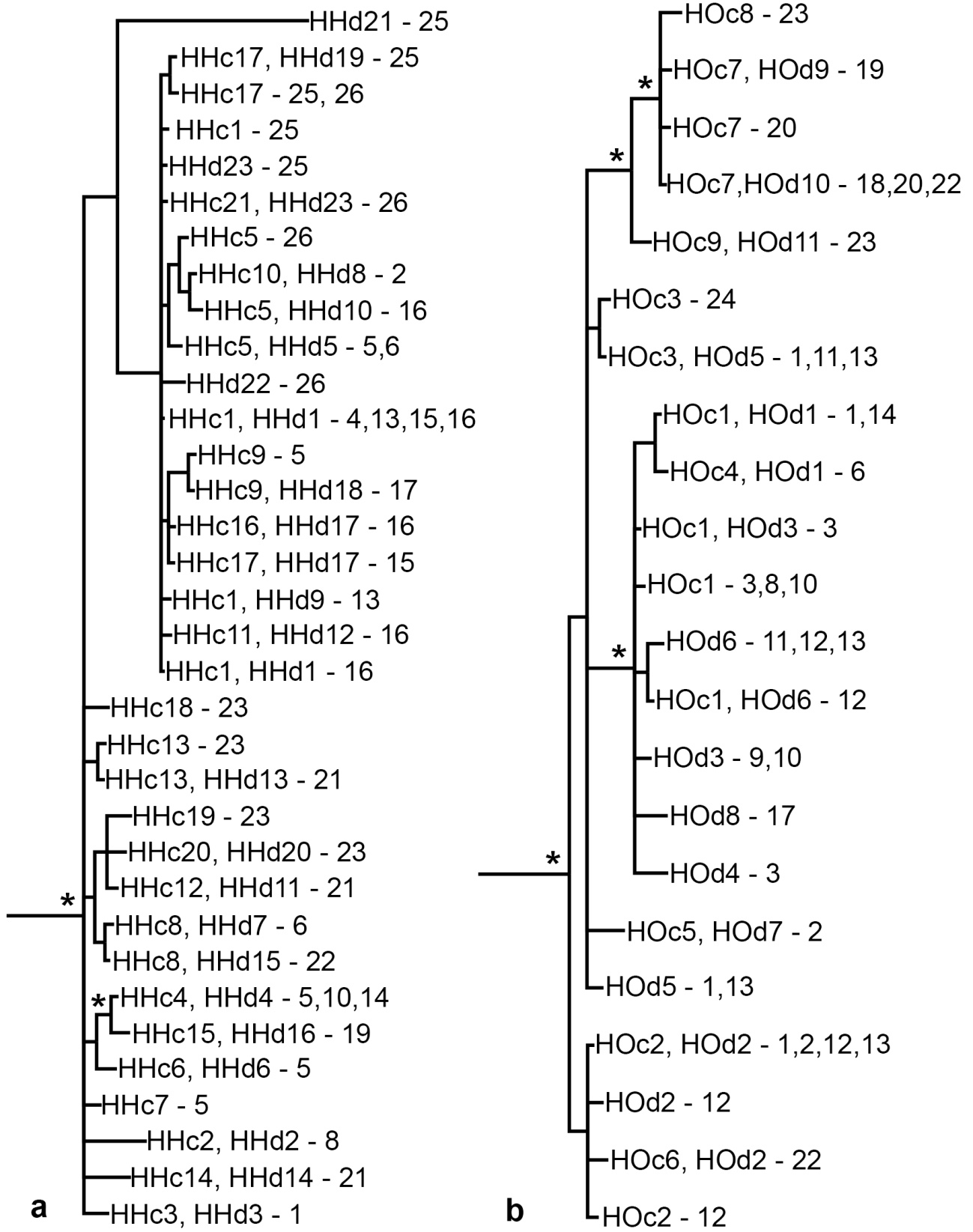
**

**Figure S1.** Bayesian inference tree of concatenated haplotypes of *Hylaeamys megacephalus* (a) and *Oecomys* aff. *roberti* (b). Asterisks indicate Bayesian posterior probabilities (BPP) ≥ 0.95; numbers in the tip of branches are sampling localities and acronyms are haplotype designations of cyt B and d-loop concatenated sequences.

**Table S1** Pairwise F_ST_ values based on cyt B sequences (below diagonal) and d-loop sequences (above diagonal) of *H. megacephalus*. F_ST_ values in bold were significantly different from zero.

|  | **1** | **2** | **4** | **5** | **6** | **8** | **10** | **13** | **14** | **15** | **16** | **17** | **19** | **21** | **22** | **23** | **25** | **26** |
| --- | --- | --- | --- | --- | --- | --- | --- | --- | --- | --- | --- | --- | --- | --- | --- | --- | --- | --- |
| **1** |  | 1.00 | 1.00 | 0.03 | -0.44 | 1.00 | 1.00 | 0.96 | 1.00 | 0.97 | 0.82 | 1.00 | 1.00 | 0.20 | 1.00 | 1.00 | -0.07 | 0.82 |
| **2** | 1.00 |  | 1.00 | -0.29 | -0.83 | 1.00 | 1.00 | 0.76 | 1.00 | 0.80 | -0.08 | 1.00 | 1.00 | 0.51 | 1.00 | 1.00 | -0.51 | 0.33 |
| **4** | 1.00 | 1.00 |  | -0.35 | -0.83 | 1.00 | 1.00 | -1.00 | 1.00 | -1.00 | -1.00 | 1.00 | 1.00 | 0.51 | 1.00 | 1.00 | -0.55 | 0.33 |
| **5** | 0.17 | -0.21 | -0.60 |  | -0.36 | 0.36 | -0.24 | **0.35** | -0.24 | 0.24 | 0.25 | -0.10 | -0.10 | 0.21 | 0.09 | 0.04 | -0.03 | 0.06 |
| **6** | -0.60 | -0.60 | -1.00 | -0.22 |  | 0.07 | -0.35 | 0.48 | -0.35 | 0.30 | 0.27 | 0.56 | -0.27 | 0.05 | -0.68 | -0.83 | -0.27 | -0.09 |
| **8** | -0.33 | 0.56 | 0.43 | 0.25 | -0.20 |  | 1.00 | 0.97 | 1.00 | 0.98 | 0.88 | 1.00 | 1.00 | 0.52 | 1.00 | 1.00 | 0.28 | 0.88 |
| **10** | 1.00 | 1.00 | 1.00 | -0.08 | -0.33 | 0.20 |  | 0.96 | 0.00 | 0.97 | 0.83 | 1.00 | 1.00 | 0.13 | 1.00 | 1.00 | -0.02 | 0.83 |
| **13** | 1.00 | 1.00 | 0.00 | 0.13 | **0.47** | **0.88** | 1.00 |  | 0.96 | -0.01 | 0.04 | 0.82 | 0.96 | **0.80** | 0.96 | **0.96** | 0.21 | 0.73 |
| **14** | 1.00 | 1.00 | 1.00 | -0.08 | -0.33 | 0.20 | 0.00 | 1.00 |  | 0.97 | 0.83 | 1.00 | 1.00 | 0.13 | 1.00 | 1.00 | -0.02 | 0.83 |
| **15** | 0.90 | 0.71 | -1.00 | 0.02 | 0.13 | 0.73 | 0.89 | 0.20 | 0.89 |  | -0.15 | 0.82 | 0.97 | 0.74 | 0.97 | 0.97 | 0.08 | 0.69 |
| **16** | 0.84 | 0.45 | -1.00 | 0.09 | 0.29 | **0.76** | 0.82 | 0.00 | 0.81 | -0.04 |  | 0.26 | 0.83 | **0.70** | 0.84 | 0.82 | 0.11 | 0.39 |
| **17** | 1.00 | 1.00 | 1.00 | -0.14 | -0.33 | 0.56 | 1.00 | 1.00 | 1.00 | 0.71 | 0.54 |  | 1.00 | 0.56 | 1.00 | 1.00 | -0.38 | 0.54 |
| **19** | 1.00 | 1.00 | 1.00 | 0.07 | -0.14 | 0.43 | 1.00 | 1.00 | 1.00 | 0.89 | 0.81 | 1.00 |  | 0.20 | 1.00 | 1.00 | 0.03 | 0.84 |
| **21** | -0.29 | 0.33 | 0.15 | 0.20 | -0.05 | -0.14 | -0.17 | **0.62** | -0.17 | **0.46** | **0.56** | 0.33 | 0.15 |  | -0.12 | 0.03 | 0.19 | 0.59 |
| **22** | 1.00 | 1.00 | 1.00 | 0.17 | -1.00 | -1.00 | 1.00 | 1.00 | 1.00 | 0.90 | 0.84 | 1.00 | 1.00 | -0.30 |  | 1.00 | -0.07 | 0.83 |
| **23** | -0.23 | 0.34 | 0.16 | **0.21** | -0.01 | -0.09 | -0.15 | **0.55** | -0.15 | **0.42** | **0.51** | 0.34 | 0.16 | -0.10 | -0.23 |  | -0.12 | 0.81 |
| **25** | 0.90 | 0.73 | -0.33 | 0.10 | 0.25 | 0.76 | 0.90 | 0.39 | 0.90 | -0.33 | 0.13 | 0.73 | 0.90 | **0.51** | 0.90 | **0.46** |  | -0.22 |
| **26** | 0.73 | 0.14 | -1.00 | -0.01 | 0.00 | 0.63 | 0.71 | **0.19** | 0.71 | -0.20 | -0.05 | 0.33 | 0.71 | **0.43** | 0.73 | **0.41** | -0.09 |  |

**Table S2** Pairwise F_ST_ values based on cyt B sequences (below diagonal) and d-loop sequences (above diagonal) of *O.* aff. *roberti*. F_ST_ values in bold were significantly different from zero.

|  | **1** | **2** | **3** | **6** | **7** | **8** | **9** | **10** | **12** | **14** | **11** | **13** | **17** | **18** | **19** | **20** | **22** | **23** | **24** |
| --- | --- | --- | --- | --- | --- | --- | --- | --- | --- | --- | --- | --- | --- | --- | --- | --- | --- | --- | --- |
| **1** |  | -0.23 | 0.44 | 0.21 | 0.21 | 0.21 | 0.21 | **0.68** | **0.36** | 0.21 | -0.23 | -0.03 | 0.33 | 0.28 | 0.38 | 0.14 | 0.28 | 0.12 | - |
| **2** | -0.29 |  | 0.15 | -0.13 | -0.38 | -0.38 | -0.38 | 0.53 | 0.17 | -0.13 | -0.26 | -0.17 | -0.13 | 0.05 | 0.18 | 0.08 | 0.05 | 0.00 | - |
| **3** | 0.17 | 0.12 |  | 0.71 | -1.00 | -1.00 | -1.00 | 0.54 | 0.11 | 0.71 | 0.32 | 0.11 | 0.33 | 0.85 | 0.87 | 0.55 | 0.85 | 0.87 | - |
| **6** | 0.11 | 0.00 | 1.00 |  | 1.00 | 1.00 | 1.00 | 1.00 | 0.37 | 0.00 | 0.22 | 0.18 | 1.00 | 1.00 | 1.00 | 0.52 | 1.00 | 1.00 | - |
| **7** | -0.33 | -0.43 | 0.00 | 1.00 |  | 0.00 | 0.00 | 0.00 | -0.33 | 1.00 | -0.08 | -0.32 | 1.00 | 1.00 | 1.00 | 0.33 | 1.00 | 1.00 | - |
| **8** | 0.17 | 0.12 | 0.00 | 1.00 | 0.00 |  | 0.00 | 0.00 | -0.33 | 1.00 | -0.08 | -0.32 | 1.00 | 1.00 | 1.00 | 0.33 | 1.00 | 1.00 | - |
| **9** | - | - | - | - | - | - |  | 0.00 | -0.33 | 1.00 | -0.07 | -0.32 | 1.00 | 1.00 | 1.00 | 0.33 | 1.00 | 1.00 | - |
| **10** | 0.17 | 0.12 | 0.00 | 1.00 | 0.00 | 0.00 | - |  | **0.28** | 1.00 | **0.65** | 0.34 | 1.00 | 1.00 | 1.00 | **0.79** | 1.00 | 1.00 | - |
| **12** | -0.17 | -0.12 | -0.02 | 0.18 | -0.50 | -0.02 | - | -0.02 |  | 0.37 | 0.19 | 0.02 | 0.36 | 0.56 | 0.61 | **0.47** | 0.55 | 0.59 | - |
| **14** | -0.33 | -0.43 | 0.00 | 1.00 | 0.00 | 0.00 | - | 0.00 | -0.50 |  | 0.22 | 0.18 | 1.00 | 1.00 | 1.00 | 0.52 | 1.00 | 1.00 | - |
| **11** | 0.20 | 0.22 | 1.00 | 1.00 | 1.00 | 1.00 | - | 1.00 | 0.49 | 1.00 |  | -0.23 | 0.13 | 0.07 | 0.22 | 0.05 | 0.07 | 0.00 | - |
| **13** | -0.50 | -0.29 | 0.17 | 0.11 | -0.33 | 0.17 | - | 0.17 | -0.17 | -0.33 | 0.02 |  | -0.01 | 0.22 | 0.34 | 0.17 | 0.22 | 0.22 | - |
| **17** | - | - | - | - | - | - | - | - | - | - | - | - |  | 1.00 | 1.00 | 0.43 | 1.00 | 1.00 | - |
| **18** | 0.68 | 0.60 | 1.00 | 1.00 | 1.00 | 1.00 | - | 1.00 | 0.79 | 1.00 | 1.00 | 0.68 | - |  | 1.00 | -1.00 | 0.00 | 1.00 | - |
| **19** | 0.68 | 0.60 | 1.00 | 1.00 | 1.00 | 1.00 | - | 1.00 | 0.79 | 1.00 | 1.00 | 0.68 | - | 0.00 |  | -0.33 | 1.00 | 1.00 | - |
| **20** | 0.50 | 0.47 | 0.61 | 0.47 | 0.40 | 0.61 | - | 0.61 | **0.61** | 0.45 | 0.54 | 0.50 | - | -1.00 | -1.00 |  | -1.00 | 0.20 | - |
| **22** | 0.68 | 0.60 | 1.00 | 1.00 | 1.00 | 1.00 | - | 1.00 | 0.79 | 1.00 | 1.00 | 0.68 | - | 0.00 | 0.00 | -1.00 |  | 1.00 | - |
| **23** | 0.70 | 0.65 | 0.89 | 0.78 | 0.75 | 0.89 | - | 0.89 | **0.79** | 0.78 | 0.87 | 0.71 | - | -1.00 | -1.00 | -0.20 | -1.00 |  | - |
| **24** | -0.60 | -0.25 | 1.00 | 1.00 | 1.00 | 1.00 | - | 1.00 | 0.31 | 1.00 | 0.00 | -0.60 | - | 1.00 | 1.00 | 0.38 | 1.00 | 0.75 |  |

**Gazetteer**

List 26 collecting localities of *Oecomys* aff. *roberti* and *Hylaeamys megacephalus*, sorted by Brazilian states. Geographic coordinates (longitude / latitude) are in negative (south) decimal degrees. Numbers correspond to the map on Figure 1.

**Tocantins (TO): 1.** Rio Coco, Parque Estadual do Cantão, Caseara -50.00/-9.39; **2.** Rio Coco, Parque Estadual do Cantão, Caseara -49.98/-9.37; **3.** Rio Coco, Parque Estadual do Cantão, Caseara -49.97/-9.43; **4.** Rio Javaés, Parque Estadual do Cantão, Pium -50.03/-9.98; **5.** Rio Javaés, Parque Estadual do Cantão, Pium -50.04/-9.98; **6.** Rio Javaés, Parque Estadual do Cantão, Pium -50.07/-9.97; **7.** Rio Javaés, Parque Estadual do Cantão, Pium -50.10/-9.97; **8.** Rio Javaés, Parque Estadual do Cantão, Pium -50.12/-9.96; **11.** Margem direita do Rio Araguaia, Parque Estadual do Cantão, Pium -50.09/-9.50; **12.** Ilha do Araguaia, Parque Estadual do Cantão, Pium -50.10/-9.47; **13.** Margem direita do Rio Araguaia, Parque Estadual do Cantão, Pium -50.09/-9.47; **14.** Ilha do Araguaia -50.15/9.74; **17.** Margem direita do Rio Araguaia, Parque Estadual do Cantão, Pium -50.14/-9.65; **18.** Ipucas, Fazenda Lago Verde, Lagoa da Confusão -49.70/-10.89; **19.** Ipucas, Fazenda Lago Verde, Lagoa da Confusão -49.71/-10.86; **20.** Ipucas, Fazenda Lago Verde, Lagoa da Confusão -49.69/-10.88; **21.** Ipucas, Fazenda Lago Verde, Lagoa da Confusão -49.69/-10.88; **22.** Ipucas, Fazenda Lago Verde, Lagoa da Confusão -49.73/-10.88; **23.** Peixe -48.64/-11.84; **24.** Palmas -48.36/-10.21.

**Pará (PA): 9.** Margem esquerda do Rio Araguaia, Fazenda Santa Fé, Santana do Araguaia -50.15/-9.63; **10.** Margem esquerda do Rio Araguaia, Fazenda Santa Fé, Santana do Araguaia -50.14/-9.63; **15.** Margem esquerda do Rio Araguaia, Fazenda Santa Fé, Santana do Araguaia -50.18/-9.74; **16.** Margem esquerda do Rio Araguaia, Fazenda Santa Fé, Santana do Araguaia -50.16/-9.68.

**Mato Grosso (MT): 25.** Barra do Garças -52.37/-15.58; **26.** Ribeirão Cascalheira -51.93/-12.64.
