# Supplementary figures and images for: Seasonal flooding regime and ecological traits influence genetic structure of two small rodents
Source: Ecol Evol. 2014 Nov 30;4(24):4598–608. doi: 10.1002/ece3.1336 (PMC4278813; doi:10.1002/ece3.1336)

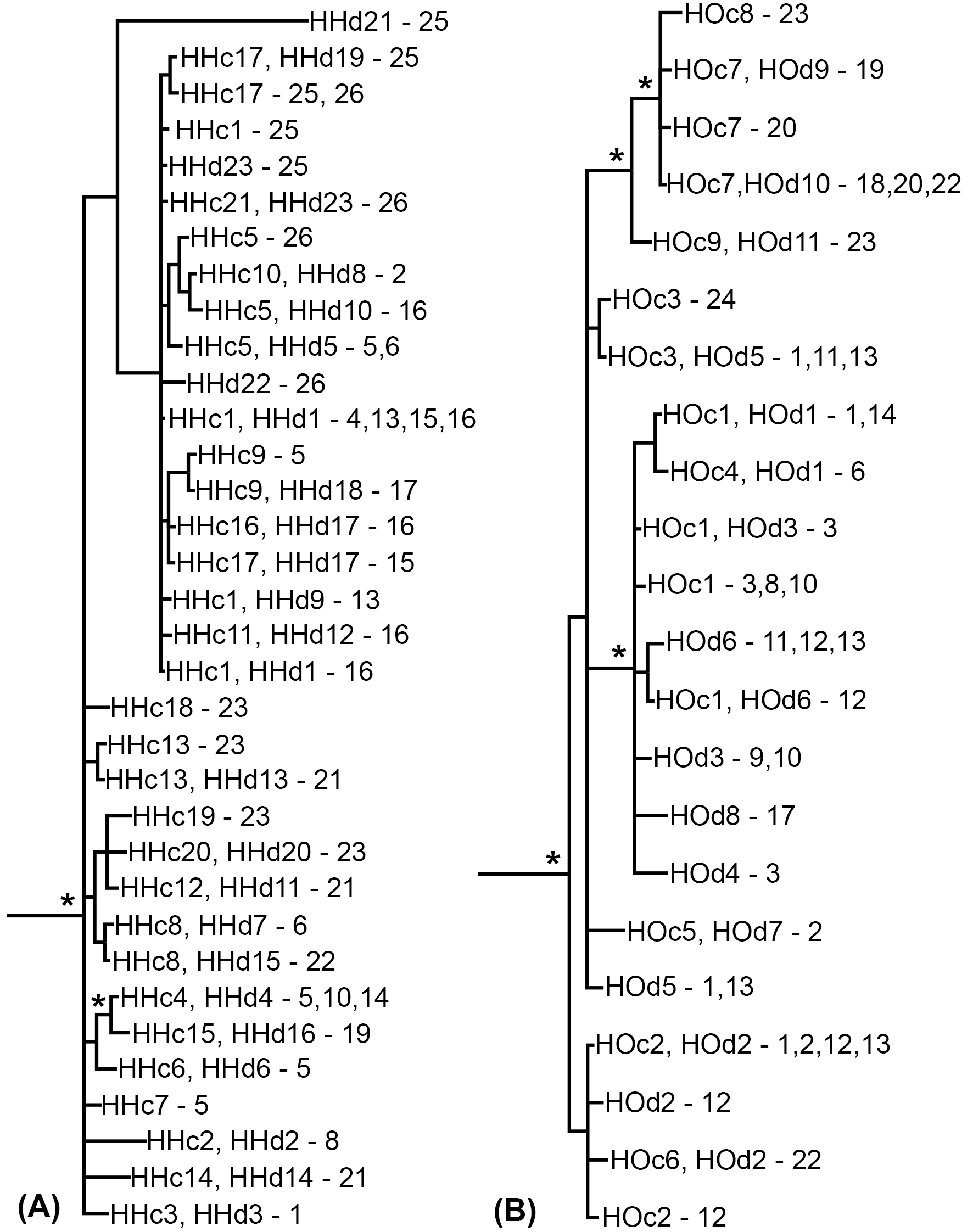

Supplement: Supplementary file 2 [file ece30004-4598-sd2.tif]
